# Effectiveness and cerebral responses of multi-points acupuncture for primary insomnia: a preliminary randomized clinical trial and fMRI study

**DOI:** 10.1186/s12906-020-02969-6

**Published:** 2020-08-17

**Authors:** Yu-Kai Wang, Tie Li, Li-Juan Ha, Zhong-Wen Lv, Fu-Chun Wang, Zhi-Hong Wang, Jing Mang, Zhong-Xin Xu

**Affiliations:** 1 Department of Neurology, China-Japan Union Hospital of Jilin University, Changchun, Jilin 130033 P.R. China; 2 Department of Acupuncture and Moxibustion, Changchun University of Chinese Medicine, Changchun, Jilin 130117 P.R. China; 3 Department of Radiology, China-Japan Union Hospital of Jilin University, Changchun, Jilin 130033 P.R. China; 4 The ‘973’ National Basic Research Program of China, Changchun University of Chinese Medicine, Changchun, Jilin 130117 P.R. China

**Keywords:** Primary insomnia, Acupuncture, Functional magnetic resonance imaging, Polysomnography

## Abstract

**Background:**

Primary insomnia (PI) is characterized by difficulties in initiating sleep or maintaining sleep, which lead to many serious diseases. Acupuncture for PI has drawn attention with its effectiveness and safety. However, the operation of choosing acupoints lacks scientific suggestion. Our trial aims to provide reference and scientific basis for the selection of acupoints and to explore its possible mechanism.

**Methods:**

A patient-assessor-blinded, randomized and sham controlled trial was designed to compare the efficacy of 5-weeks acupuncture at a single acupoint, the combination of multi-acupoints, and a sham point. The Pittsburgh sleep quality index and Athens Insomnia Scale questionnaire were used for the primary clinical outcomes, while polysomnography was performed for the secondary clinical outcomes. The resting state functional MRI was employed to detect the cerebral responses to acupuncture. The brain activity in resting state was measured by calculating the fractional amplitude of low-frequency fluctuations (fALFF), which reflected the idiopathic activity level of neurons in the resting state. These results were analyzed by two factorial ANOVA test and post-hoc t-tests.

**Results:**

The clinical outcomes suggest that acupuncture could improve clinical symptoms, and the combination of multi-acupoints might lead to a better clinical efficacy. The rs-fMRI results suggested that the brain activity of certain regions was related to the sleep experience, and acupuncture could regulate the activity of these regions. Furthermore, the combination of multi-acupoints could impact more regions which were influenced by the sleep experience.

**Conclusions:**

Acupuncture has been proven to be beneficial for PI patients, and the combination of multi-acupoints might improve its efficacy.

**Trial registration:**

This trial has been registered on the U.S. National Library of Medicine (https://clinicaltrials.gov) ClinicalTrials.gov Identifier: NCT02448602. Registered date: 14/04/2015.

## Background

Primary insomnia (PI) is defined as a symptom of prolonged sleep latency and difficulty in maintaining sleep [1]. As a prevalent sleep disorder in the global population with rising prevalence [2], PI has caused an increased incidence of serious medical conditions, such as obesity, heart disease, high blood pressure and diabetes [3]. In general, treatments for PI include nonpharmacologic and pharmacologic therapies. However, pharmacological therapies remain limited due to potential risks and barriers, including long-term usage and the range of undesirable side-effects [4]. As a result, the exploration for more convenient treatments with less side-effect for PI patients has continued for decades.

Acupuncture, which is a technique of inserting needles into particular points of body, has been proven to be effective for insomnia with minor side-effects [5, 6]. Hence, acupuncture has been increasingly used in private clinics located in 46 states in the United States as a treatment for insomnia [7]. However, there is no standard regimen for acupuncture in the clinical treatment of insomnia, especially in the selection of acupoints. In general, acupuncturists could choose single acupoint or a combination of multi-acupoints with the same efficacy to treat insomnia. Although this is the main factor that affects efficacy, it remains unclear how the number of stimuli acupoints interfere with clinical efficacy. Furthermore, the mechanism of different efficacy produced by different acupuncture scheme remained unclear. Previous studies on the mechanism of acupuncture have revealed that acupuncture can affect the activation in brain regions through needling/tactile somatosensory specific stimuli [8–10]. Further, acupuncture might have lasting and strong sustained effects on cerebral functional regions, which was founded in a study, that is, the activation in brain regions at 15 min after acupuncture stimuli were greater than the activation at 5 min after acupuncture stimuli [11].

In order to determine which kind of acupoints scheme (a single acupoint or a combination of multi-acupoints) could produce better efficacy, a patient-assessor-blinded, randomized and sham controlled trial was designed for PI patients. In the present study, PI patients randomly accepted electro-acupuncture stimulation on single acupoint, multi-acupoints, sham points for 5 weeks. Pittsburgh sleep quality index (PSQI), Athens Insomnia Scale (AIS) questionnaires and the polysomnography (PSG) were performed to evaluate the sleep condition at baseline and after the treatment. Resting state functional magnetic resonance imaging (rs-fMRI) could detect blood oxygenation level dependent (BOLD) signals in brain in resting state [12]. Amplitude of low-frequency fluctuations (ALFF) could directly reflect idiopathic activity levels of neurons in the voxels according to the BOLD signals [13]. Furthermore, a modified calculation called fractional amplitude of low-frequency fluctuation (fALFF), which means the ratio of the power spectrum of low frequency (0.01–0.08 Hz) to that of the entire frequency range, was developed to suppress non-specific noise components [14]. Taken together, the present study aims to provide a reference and scientific basis for the choice of acupoints in clinical treatment, and expect to be helpful in improving the clinical efficacy of acupuncture for PI patients.

## Methods

### Subjects

The present study is a patient-assessor-blinded, randomized and sham controlled trial (No. NCT02448602, registered on 14/04/2015). From September 2015 to September 2018, PI patients were recruited from outpatient clinics in the Neurological Department of China-Japan Union Hospital of Jilin University and Changchun University of Chinese Medicine. All subjects satisfied the following criteria. Inclusion criteria [15]: (1) patients who were over 18 years old, but under 65 years old; (2) patients who had no problems with communication and intelligence; (3) patients with a wake after sleep onset (WASO) or sleep onset latency (SOL) of > 30 min for at least 3 nights every week, and symptoms lasting for over 3 months; (3) patients with an AIS score of ≥6; (4) patients with a PSQI score of > 7. Exclusion criteria [15]: (1) patients with uncontrolled medical conditions suspected to interfere with sleep; (2) patients with uncontrolled psychiatric conditions requiring immediate treatment; (3) patients diagnosed of comorbid sleep disorders; (4) patients with a self-rating depression scale (SDS) or self-rating anxiety scale (SAS) of ≥50; (5) patients with alcohol and/or other drug abuse or dependence; (6) Pregnant or nursing patients, or those of childbearing age who have not adopted appropriate birth control methods; (7) patients with who took hypnotic or sedating medications, or accepted acupuncture treatment in the recent 1 month.

The participants were informed that the intervention in this trial included three different acupuncture regimens (but they did not know the acupoints of other regimens except the regimens they accepted in the course of the trial), the treatment efficacy might be different, the patients would randomly accept one acupuncture regimens. Patients were included in the trial after approval and signature of informed consent (Permission number: CCZYFYLL2014–043).

### Acupuncture procedures

**Fig. 1:**
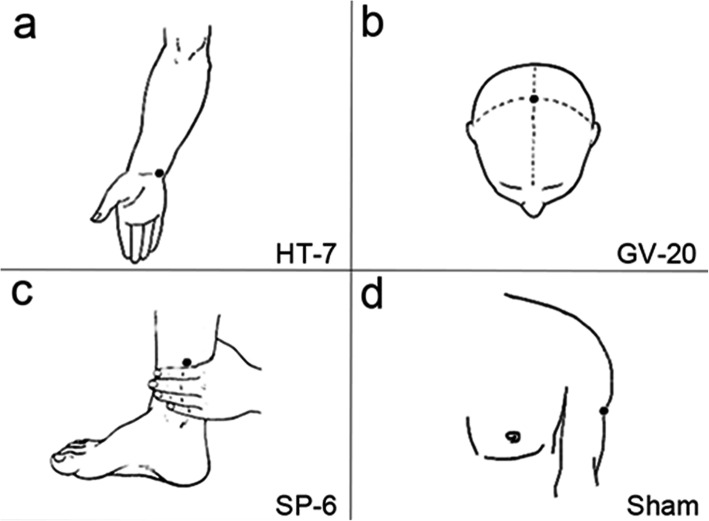
The location of the acupoints and diagrammatic sketch of the fMRI scan process. **a** The location of the acupoint in S-Acu group. **a**-**c** The location of acupoints in M-Acu group. **d** The location of the sham point in N-Acu group

The acupoints used in this trial are Shenmen (HT-7), Sanyinjiao (SP-6), Baihui (GV-20), which are recommended points for insomnia treatment [5, 6, 16]. HT-7 is the point with the highest frequency of use [17], SP-6 and GV-20 are often used with HT-7 as combination regimen according to the Traditional Chinese Medicine (TCM) theory and clinical experience of acupuncture therapists [18]. Also, a sham point used as control group to exclude the placebo effect of acupuncture. The patients were randomly divided into three groups, and accepted electro-acupuncture for 5-weeks on different acupoints: single acupoints group (S-Acu group, bilateral HT-7) (Fig. 1a); multi-acupoints group (M-Acu group, combination of bilateral HT-7, bilateral SP-6 and single GV-20) (Fig. 1a-c); Non-acupoint group (N-Acu group, bilateral sham point at the junction point between the biceps brachii muscle and deltoid muscle) (Fig. 1d). The randomized achieve through the network system of Clinical Evaluation Center of China Academy of Chinese Medical Sciences. During the acupuncture, a 25 × 0.35 mm sterile and reusable acupuncture silver needles was inserted into the bilateral points at a depth of 10 mm [18] (the GV-20 was at a depth of 5–8 mm [19]). Another silver needle was inserting at a distance of 1 cm from the acupoint. The handle of these two needles were connected to an electro-acupuncture machine (Suzhou Medical Appliance Factory, China) with a frequency of 4 Hz [20] and an intensity that maintained the De-Qi sensation [21]. The Massachusetts General Hospital acupuncture sensation scales (MASS) was used to determine whether the De-Qi sensations was produced during the acupuncture. The intensity of soreness, numbness, heaviness, warmth, cold, sharp pain and dull pain were rate on a numerical scale of 1–10 to screen patients with the De-Qi sensation. The acupuncture treatment lasted for 5 weeks, five times per week, and 30 min each time.

### Observation of clinical efficacy

All patients accomplished the AIS and PSQI questionnaires at baseline and at the end of the 5-week treatment. The AIS questionnaire is a self-administered psychometric instrument that consisted of eight items. Each item was rated from 0 to 3, for a total score range of 0–24. A score of “0” indicates no problem at all, while a score of “24” indicates very serious problems in all areas [22]. The PSQI questionnaire consists of 19 self-rated questions, which are grouped into seven component scores, each ranging from 0 to 3. Then, the seven component scores are summed to yield a global PSQI score, which has a range of 0–21, with higher scores indicating worse sleep quality [23].

Patients underwent the polysomnography (PSG, Embletta X100) assessment at baseline and the end of the 5-weeks treatment. The PSG test was performed according to American Academy of Sleep Medicine (AASM) standards after an adaption night in single rooms. The duration of the PSG recording was 480 min. The PSG monitor physiologic data during the sleep cycle included the electrooculogram (EOG), electroencephalogram (EEG), electrocardiogram (ECG), electromyogram (EMG) (submental and bilateral masseter), airflow measurements using both oronasal-thermal sensors and nasal air pressure transducers, rib cage and abdominal movement by inductance plethysmography using thoracoabdominal belts, and continuous pulse oximetry. Variables, including total sleep time (TST), sleep efficient (SE), sleep stages (stage NREM1 (N1), stage N2, stage N3, and stage REM), wake after sleep onset (WASO) and arousal index (AI), were analyzed with the above-mentioned PSG data.

### Statistical analysis of clinical outcomes

The clinical data were analyzed using SPSS 18.0 statistical software. The demographic characteristic of three groups including gender, age, SAS and SDS were analyzed with one-way ANOVA test. The MASS scale of three groups were analyzed with one-way ANOVA test. The primary and secondary clinical outcomes, the AIS / PSQI scores and the PSG data at baseline and after the treatment, were analyzed with a two factorial ANOVA testing. The change of clinical outcomes among three group were analyzed with ANOVA analyses and post-hoc t-tests. All data were expressed as mean ± standard deviation.

### FMRI data acquisition

We stratified the patients in the S-Acu group who agreed to accept fMRI examination according to the age of 18 ~ 35, 36 ~ 50, 51 ~ 65, and randomly selected 5 patients from each age group, a total of 15 patients received fMRI examination. The same were the M-Acu group and the N-Acu group. PI patients received rs-fMRI assessment at baseline and 1 day after 5 weeks of acupuncture treatment between 8:00 a.m. and 10:00 a.m. during which the subjects were relatively awake.

The fMRI scan was completed on a 3.0 T whole-body MRI scanner (MAGNETOM-Skyra-SIEMENS) with a volume transmit head coil and 32-channel receive coil. The MRI sequences included the following: (1) T1-weighted MRI: data were acquired using a magnetization-prepared rapid gradient-echo sequence with TR/TE at 700 ms/11 ms, FOV at 256 × 256 × 192, and a voxel size of 1 × 1 × 0.9; (2) rs-fMRI: data were acquired using an echo planar imaging (EPI) sequence sensitive to BOLD with TR/TE/FA at 2020 ms/30 ms/90°, and FOV at 106 × 106 × 46, and a voxel size of 2.4 × 2.4 × 3.75. The rs-fMRI scan duration lasted 200 TR [15].

### Rs-fMRI data processing

The main preprocessing steps of rs-fMRI included the following [15]: Digital Imaging and Communications in Medicine (DICOM) data were converted into NIFTI data; the first 10 volumes were discarded for signal stabilization and subject adaptation; then, slice timing, spatial realignment, head motion correction, individual registration between high-resolution T1 and echo planar imaging (EPI) images, T1 segmentation with the Diffeomorphic Anatomical Registration Through Exponentiated Lie algebra (DARTEL) and spatial normalization to register rs-fMRI data sets to the Montreal Neurological Institute (MNI) space were performed, along with resampling to 3 × 3 × 3 mm^3^ cube voxels; and head motion estimation, > 1.5 mm of the maximal translation or 1.5° of the maximal rotation, was excluded from the final analysis. Then the normalized data were spatially smoothed using a 6 mm full-width half-maximum Gaussian kernel. Linear detrending and nuisance linear regression (including the white matter, the cerebrospinal fluid and head motion parameters) were performed, and a temporal bandpass filter (0.01–0.08 Hz) was applied to reduce the effects of head motion and nonneuronal BOLD fluctuations [24]. ALFF and fALFF were calculated with DPARSF package for each subject [14].

This preprocessing was performed using the Data Processing Assistant for Resting-State fMRI (DPARSF) package (http://www.restfmri.net) [24] based on statistical parametric mapping (SPM8, Welcome Department of Imaging Neuroscience, Institute of Neurology, London; http://www.fil.ion.ucl.ac.uk/spm), which was run on MATLAB (MathWorks, Natick, MA, USA).

### Rs-fMRI data analysis

SPM8 was employed for the rs-fMRI data analysis. First, the relationship between the clinical outcome and brain activity were studied. The fALFF value of PI patients was analyzed by multiple regression analysis with AIS score as covariable (*P* < 0.001, FWE correct *P* = 0.05, cluster size > 30).

The altered fALFF value after treatment of three groups were analyzed by one-way ANOVA with-in subject test (flexible factorial model) with the groups (S-Acu group, M-Acu group, N-Acu group) as the between-subject factor and the repeated measure (baseline and after treatment) as the within-subjects factor (*P* < 0.001, FWE correct *P* = 0.05, cluster size> 30). Furthermore, the difference of S-Acu group vs N-Acu group, and S-Acu group vs M-Acu group were analyzed with post-hoc t-tests (*P* < 0.005, FWE correct *P* = 0.05, cluster size> 30). All results were presented by the REST v1.8 software [25].

### Patient safety

Any adverse events related to the acupuncture treatment, including unfavorable or unintended signs, symptoms, or diseases occurring after treatment, were observed and reported. The patients will immediately terminate the trial if adverse event happened.

### Quality control

One investigator and an acupuncturist participated in the present study, and they both received special training before the trial, in order to ensure consistency in practice. The training program included the following: diagnosis, inclusion and exclusion criteria, case reports, location of the acupoints, and acupuncture manipulation techniques. The investigator did not know the groupings, the group assigned for each patient, and the person responsible for the evaluation of patients and analysis of all data before and after the acupuncture treatment. The acupuncturist, who has been engaged in clinical acupuncture for more than 3 years, was responsible for the administration of treatment and the operation of the acupuncture. All test results were reviewed and adjudicated by a professional neurologist, who did not know which group the patient’s result belonged to, or who responsible for the evaluation of clinical data. The patients were not informed of the treatment for other groups, except for the treatment they were receiving. The accuracy and quality of the whole study was ensured through regular monitoring during the intervention periods.

## Results

### Demographic characteristic in different groups

In our trials, 129 patients (mean age: 52.66 ± 11.14 years old, 27 males) were included in the final analysis. The subjects were randomly divided into 3 groups (S-Acu group: *n* = 43; M-Acu group: n = 43; N-Acu group: n = 43). There were no significant differences in gender, age, PSQI, AIS, SAS and SDS scores among the three groups (*P* > 0.05) (Table 1). And the type of needling sensations was recorded with MASS scale. There were no significant differences among the intensity of seven sensations (*P* > 0.05) (Table 2).

**Table 1:** Demographic characteristic at baseline in different groups

Parameter	S-Acu group (HT-7)	M-Acu group (HT-7, GV-20 and SP-6)	N-Acu group (sham point)
Sex			
Male	11	9	7
Female	32	34	36
Age(years)	55.42 ± 9.23	51.45 ± 12.10	51.10 ± 11.60
PSQI	14.70 ± 2.99	14.84 ± 2.77	15.21 ± 2.95
AIS	12.40 ± 3.14	13.26 ± 4.21	13.56 ± 4.48
SAS	38.95 ± 3.96	40.19 ± 5.75	38.56 ± 7.40
SDS	36.86 ± 7.01	36.12 ± 6.72	34.70 ± 8.59

*PSQI* Pittsburgh sleep Quality index, *AIS* Athens Insomnia Scale, *SAS* self-rating anxiety scale, *SDS* self-rating depression scale, *S-Acu group* single acupoint group, *M-Acu group* multi-acupoints group, *N-Acu group* non-acupoint group

**Table 2:** Comparisons of the intensity of De-Qi sensations among the three groups

Sensations	S-Acu group (HT-7)	M-Acu group (HT-7, GV-20 and SP-6)	N-Acu group (sham point)	P
soreness	1.74 ± 0.76	2.02 ± 0.77	1.93 ± 0.74	0.223
numbness	1.74 ± 0.69	1.91 ± 0.68	1.79 ± 0.68	0.526
heaviness	2.74 ± 0.76	2.98 ± 0.80	2.77 ± 0.78	0.318
warmth	1.51 ± 0.74	1.58 ± 0.79	1.47 ± 0.74	0.773
cold	0.74 ± 0.76	0.63 ± 0.72	0.70 ± 0.74	0.766
sharp pain	2.74 ± 0.76	2.93 ± 0.80	2.86 ± 0.74	0.526
dull pain	2.56 ± 0.67	2.65 ± 0.65	2.58 ± 0.66	0.794

*S-Acu group* single acupoint group, *M-Acu group* multi-acupoints group, *N-Acu group* non-acupoint group

### Primary clinical outcomes in different groups

After the 5-week acupuncture treatment, the AIS score and PSQI score were significantly decreased in all groups (*P* < 0.001). There were significant differences in AIS (*P* = 0.025) / PSQI scores (*P* = 0.001) among the three groups after acupuncture treatment. So, one-way ANOVA and post-hoc t-tests were performed for the decreased AIS and PSQI score of three groups. The decreased AIS score of M-Acu group was significantly higher than that of N-Acu group (*P* = 0.026). And the decreased PSQI score of S-Acu group (*P =* 0.009) and M-Acu group (*P =* 0.004) were significantly higher than that of N-Acu group (Table 3).

**Table 3:** The AIS and PSQI scores at baseline and after acupuncture treatment in different groups

	S-Acu group (HT-7)	M-Acu group (HT-7, GV-20 and SP-6)	N-Acu group (sham point)
AIS			
Baseline	12.40 ± 3.14	13.26 ± 4.21	13.56 ± 4.48
Post-treatment	7.00 ± 3.90^a^	5.91 ± 3.92^a^	8.88 ± 3.36^a^
Decrease	5.40 ± 4.63	7.35 ± 3.85^b^	4.67 ± 4.52
PSQI			
Baseline	14.70 ± 2.99	14.84 ± 2.77	15.21 ± 2.95
Post-treatment	8.79 ± 3.18^a^	8.49 ± 3.36^a^	11.60 ± 3.54^a^
Decrease	5.91 ± 3.03^c^	6.35 ± 3.39^c^	3.60 ± 3.63

^a^*P* < 0.01 (compared with baseline); ^b^*P* < 0.05 (compared with N-Acu group); ^c^*P* < 0.01 (compared with N-Acu group). *S-Acu group* single acupoint group, *M-Acu group* multi-acupoints group, *N-Acu group* non-acupoint group

### Secondary clinical outcomes in the different groups

At baseline, there were no significant difference in the WASO (*P* = 0.448), TST (*P* = 0.271), SE (*P* = 0.381), AI (*P* = 0.055), N1 (*P* = 0.305), N2 (*P* = 0.368), N3 (*P* = 0.373), R (*P* = 0.890) among three groups. After 5-weeks acupuncture, the TST (*P* = 0.020), R (*P* = 0.001) of S-Acu group were significant changed. The WASO (*P* = 0.004), TST (*P* < 0.001), SE (*P* = 0.001), AI (*P* = 0.009), N3 (*P* = 0.001), R (*P* = 0.017) of M-Acu group were significant changed. However, there was no significant difference was found in N-Acu group after 5-weeks acupuncture. There were significant differences in the WASO (*P* < 0.001) / TST (*P* < 0.001) / SE (*P* < 0.001) / N3 (*P* = 0.001) among three groups after acupuncture treatment (Table 4). So, the change of WASO, TST, SE, N3 in S-Acu group and M-Acu group were compared using independent two sample T-test. The results showed that the increase of TST in M-Acu group was significantly higher than that in S-Acu group (*P* = 0.042). The decrease of WASO and the increase of SE and N3 in M-Acu group were higher than those in S-Acu group, but there was no significant difference (Table 5).

**Table 4:** The PSG data at baseline and after acupuncture treatment in different groups

	S-Acu group		M-Acu group		N-Acu group	
	Baseline	Post-treatment	Baseline	Post-treatment	Baseline	Post-treatment
WASO (minutes)	198.58 ± 89.64	179.06 ± 76.58	203.57 ± 73.54	154.84 ± 91.19^a^	214.24 ± 68.41	228.27 ± 67.80
TST (minutes)	248.05 ± 91.77	288.25 ± 76.51^b^	247.50 ± 76.03	319.90 ± 92.51^a^	236.80 ± 72.25	247.56 ± 66.50
SE (%)	55.44 ± 20.39	61.70 ± 16.25	54.79 ± 16.66	67.34 ± 19.33^a^	52.37 ± 15.53	52.06 ± 14.12
AI	27.12 ± 12.72	21.51 ± 11.01	27.58 ± 10.77	21.16 ± 9.71^a^	26.77 ± 10.34	22.86 ± 11.57
N1 (minutes)	40.80 ± 30.49	38.41 ± 22.61	36.94 ± 20.67	38.27 ± 24.70	37.49 ± 25.97	35.37 ± 23.34
N2 (minutes)	62.21 ± 49.88	60.64 ± 52.78	60.53 ± 63.38	76.99 ± 68.21	52.45 ± 53.97	58.77 ± 47.42
N3 (minutes)	74.99 ± 48.69	85.64 ± 48.11	66.98 ± 45.07	98.26 ± 47.43^a^	69.80 ± 36.80	63.08 ± 33.05
R (minutes)	70.05 ± 45.01	103.56 ± 44.18^a^	83.05 ± 52.23	106.38 ± 44.79^b^	77.06 ± 41.47	90.34 ± 42.93

^a^*P* < 0.01 (compared with baseline); ^b^*P* < 0.05 (compared with baseline); *WASO* wake after sleep onset, *TST* total sleep time, *SE* sleep efficient, *AI* arousal index, *N1* first stage of non-rapid eye movement sleep, *N2* second stage of non-rapid eye movement sleep, *N3* third stage of non-rapid eye movement sleep, *R* sleep stage of rapid eye movement, *S-Acu group* single acupoint group, *M-Acu group* multi-acupoints group, *N-Acu group* non-acupoint group

**Table 5:** The comparison of significant varied PSG data between S-Acu group and M-Acu group

	S-Acu group (HT-7)	M-Acu group (HT-7, GV-20 and SP-6)
The increase of TST (minutes)	40.20 ± 55.48	72.40 ± 85.74^a^
The increase of SE (%)	6.26 ± 12.44	12.55 ± 18.65
The increase of N3 (minutes)	10.65 ± 48.61	31.28 ± 50.45
The decrease of WASO (minutes)	19.53 ± 54.19	48.73 ± 87.83

^a^*P* < 0.05 (compared with S-Acu group); *TST* total sleep time, *SE* sleep efficient, *N3* third stage of non-rapid eye movement sleep, *WASO* wake after sleep onset, *S-Acu group* single acupoint group, *M-Acu group* multi-acupoints group

### Rs-fMRI results in different groups

#### The relationship between the fALFF value of brain regions and AIS scores of PI

We found a negative correlation between fALFF and AIS scores in the following brain regions: right cerebellum posterior lobe, temporal gyrus (left middle temporal gyrus, left superior temporal gyrus), left extra-nuclear, right anterior cingulate, left parahippocampa gyrus, frontal gyrus (bilateral inferior frontal gyrus, left middle frontal gyrus, bilateral superior frontal gyrus), parietal lobule (right precentral gyrus, left postcentral gyrus, left supramarginal gyrus, left inferior parietal lobule, left superior parietal lobule, left precuneus) (*P* < 0.001, FWE correct *P* = 0.05, cluster size> 30) (Table 6).

**Table 6:** The relationship between the fALFF value of brain regions and AIS scores of PI

Brain regions	BA	Side	Cluster size	MNI			t-value
				X	Y	Z	
Cerebellum Posterior Lobe		R	37	9	−42	−60	−5.42
Middle Temporal Gyrus		L	34	−57	− 57	−3	−6.25
Middle Temporal Gyrus	22	L	217	−57	−42	0	−7.53
Superior Temporal Gyrus							
Parahippocampa Gyrus		L	36	−24	−27	−27	−5.56
Extra-Nuclear		L	79	−21	27	0	−5.67
Anterior Cingulate		R	345	9	30	18	−5.81
Inferior Frontal Gyrus							
Middle Frontal Gyrus	9 6	L	1599	−24	27	27	−7.08
Inferior Frontal Gyrus							
Superior Frontal Gyrus							
Precentral Gyrus	9	R	325	24	6	27	−6.76
Inferior Frontal Gyrus							
Precentral Gyrus	6	R	54	66	−3	27	−5.03
Supramarginal Gyrus	40	L	345	−54	−48	36	−7.07
Inferior Parietal Lobule							
Postcentral Gyrus							
Superior Parietal Lobule	19	L	527	−30	−72	48	−6.15
Precuneus							
Superior Frontal Gyrus	8	R	59	24	12	51	−5.49
Superior Frontal Gyrus	6	L	55	−3	18	66	−5.89

The anatomical locations, approximate Brodmann areas (BA) and MNI coordinates that corresponded to the t-values of the representative peaks within each cluster were reported. A negative t-value means that fALFF was lower in this brain region when the AIS value is higher. All regions reached a voxel-level significance threshold of *P* < 0.001, FWE correct *P* = 0.05, cluster size> 30

#### The altered fALFF regions of PI patients after acupuncture treatment

**Fig. 2:**
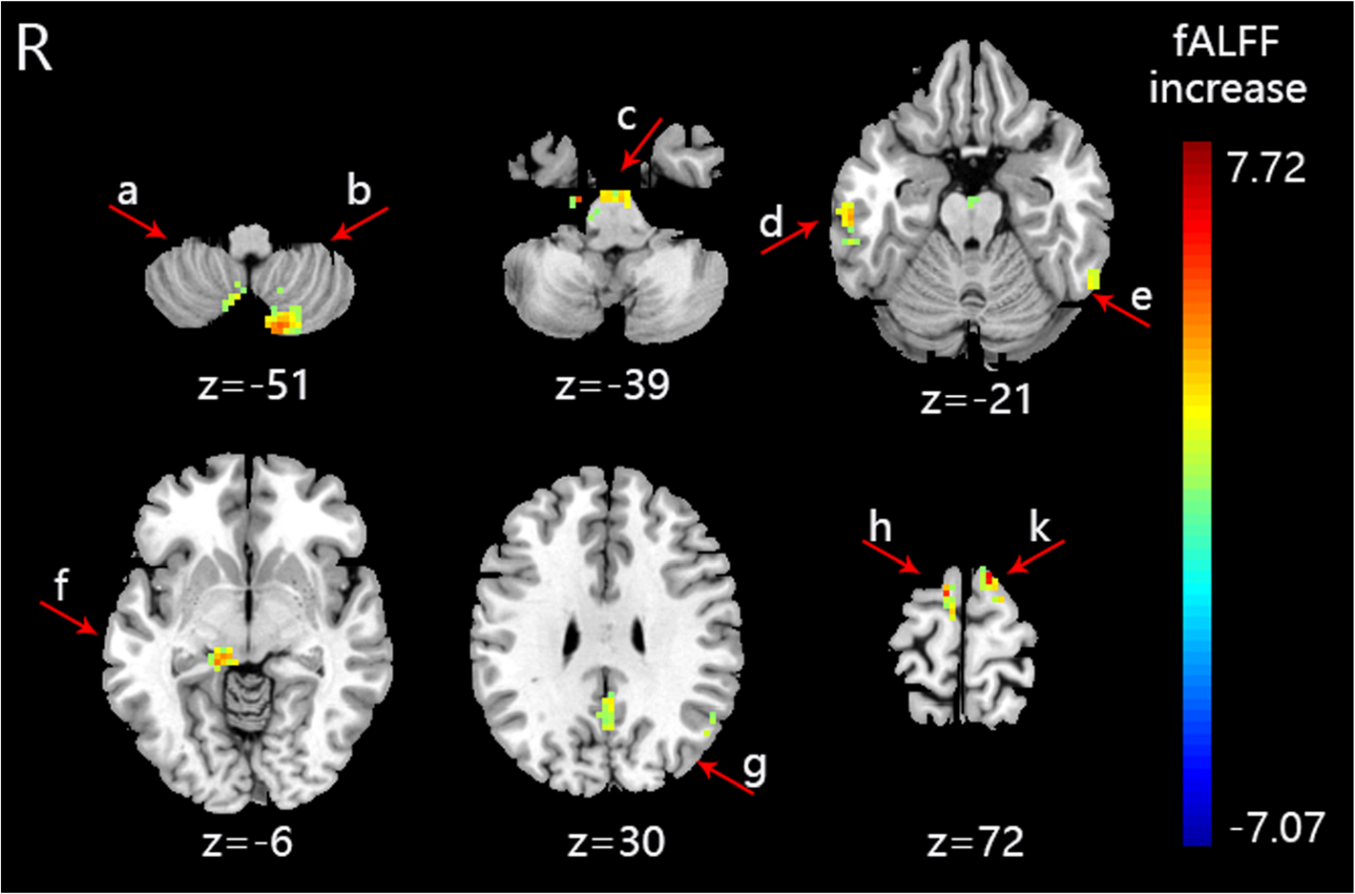
The brain regions with altered fALFF of S-Acu group after acupuncture treatment (*P* < 0.001, FWE correct *P* = 0.05, cluster size> 30). R: right brain. **a**: right cerebellum posterior lobe, **b**: left cerebellum posterior lobe, **c**: brainstem, **d**: right inferior temporal gyrus, **e**: left middle temporal gyrus, **f**: right parahippocampa gyrus, **g**: left precuneus, **h**: right superior frontal gyrus, **k**: left superior frontal gyrus. Colorbar refer to the increased fALFF value

**Fig. 3:**
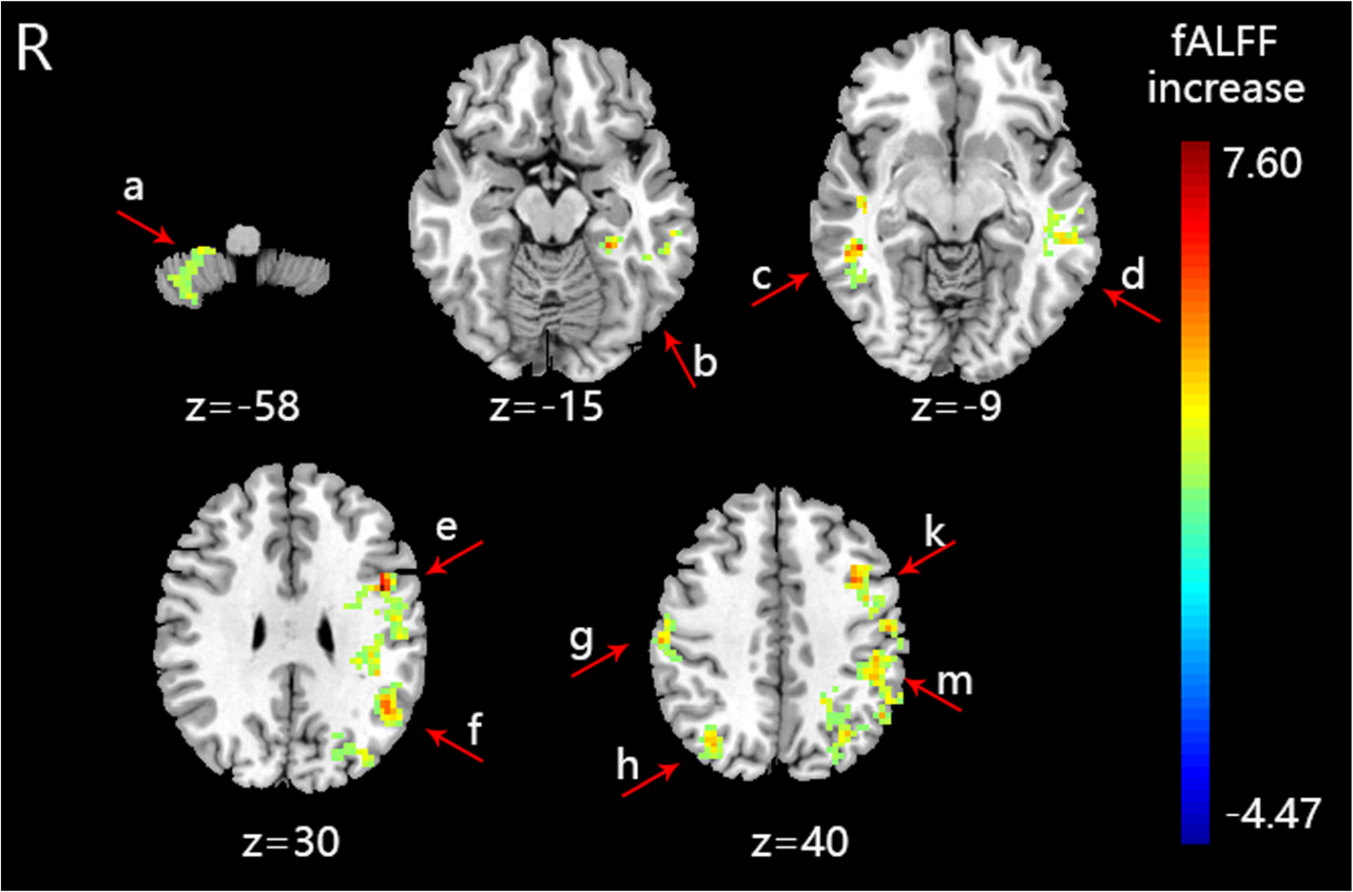
The brain regions with altered fALFF of M-Acu group after acupuncture treatment (*P* < 0.001, FWE correct *P* = 0.05, cluster size> 30). R: right brain. **a**: right cerebellum posterior lobe, **b**: left parahippocampa gyrus, **c**: left superior temporal gyrus and middle temporal gyrus, **d**: right superior temporal gyrus and middle temporal gyrus, e: left inferior frontal gyrus, **f**: left supramarginal gyrus, **g**: right precentral gyrus, **h**: right precuneus, **k**: left middle frontal gyrus, m: left postcentral gyrus. Colorbar refer to the increased fALFF value

**Fig. 4:**
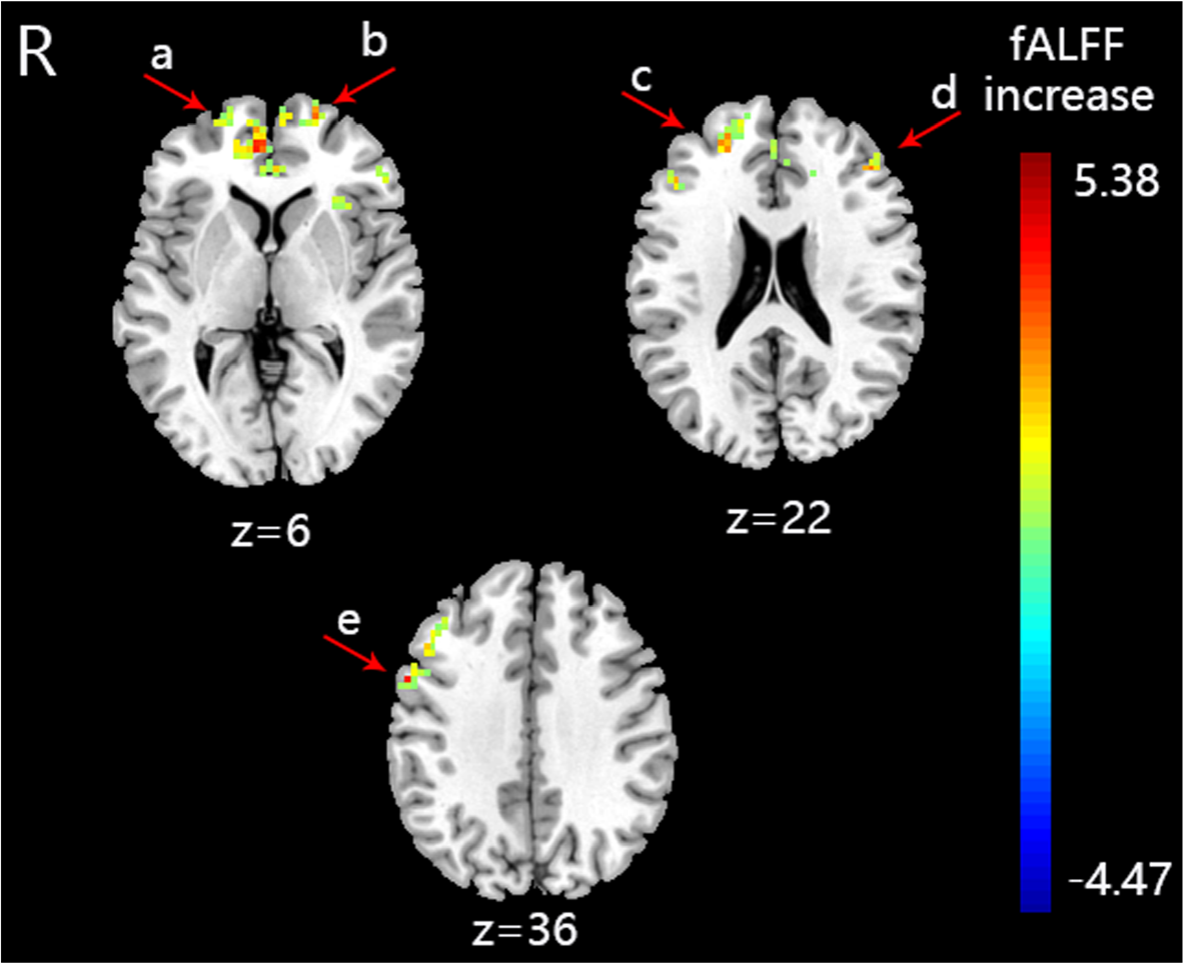
The brain regions with altered fALFF of N-Acu group after acupuncture treatment (*P* < 0.001, FWE correct *P* = 0.05, cluster size> 30). R: right brain. **a**: right superior frontal gyrus and medial frontal gyrus, **b**: left superior frontal gyrus, **c**: right middle frontal gyrus, **d**: left middle frontal gyrus, **e**: right inferior frontal gyrus. Colorbar refer to the increased fALFF value

**Fig. 5:**
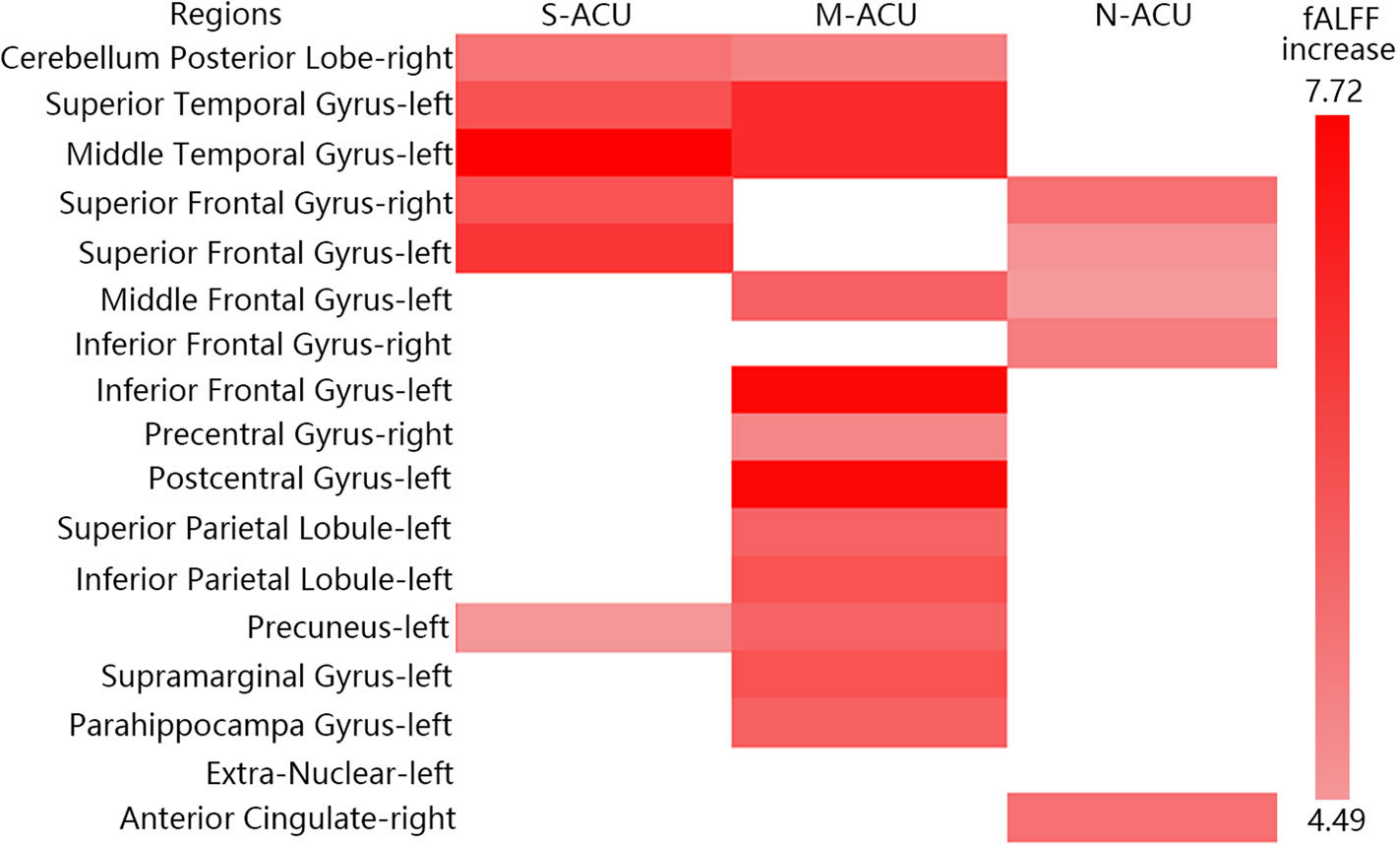
The distribution of brain regions where fALFF were negative correlation with AIS scores, and brain regions which were regulated by three acupuncture regimens. Colorbar refer to the increased fALFF value

The fALFF value of PI patients (S-Acu group: *n* = 15; M-Acu group: n = 15; N-Acu group: n = 15) were all changed after the 5-week acupuncture treatment. In S-Acu group, the fALFF of PI patients increased in bilateral cerebellum posterior lobe, bilateral brainstem, temporal gyrus (right inferior temporal gyrus, left middle temporal gyrus, left superior temporal gyrus), right parahippocampa gyrus, left precuneus, bilateral superior frontal gyrus (*P* < 0.001, FWE correct *P* = 0.05, cluster size> 30) (Table 7, Fig. 2). In M-Acu group, the fALFF of PI patients increased in right cerebellum posterior lobe, left parahippocampa gyrus, temporal gyrus (bilateral middle temporal gyrus, bilateral superior temporal gyrus), frontal gyrus (left middle frontal gyrus, left inferior frontal gyrus), parietal gyrus (bilateral precentral gyrus, bilateral postcentral gyrus, left inferior parietal lobule, left superior parietal lobule, left angular gyrus, left supramarginal gyrus, bilateral precuneus), left cuneus (*P* < 0.001, FWE correct *P* = 0.05, cluster size> 30) (Table 8, Fig. 3). In N-Acu group, the fALFF of PI patients increased in frontal gyrus (bilateral superior frontal gyrus, right medial frontal gyrus, bilateral middle frontal gyrus, right inferior frontal gyrus), right anterior cingulate, left insula (*P* < 0.001, FWE correct *P* = 0.05, cluster size> 30) (Table 9, Fig. 4). We also listed the regions where fALFF were negative correlation with AIS scores, and the regions in which could be regulated by three acupuncture regimens (Fig. 5). The results showed that the brain regions where fALFF were negative correlation with AIS scores could be partially regulated in each group, and the M-Acu group could regulate more brain regions.

**Table 7:** The altered fALFF in brain regions of S-Acu group after 5-weeks acupuncture

Brain regions	BA	Side	Cluster size	MNI			t-value
				X	Y	Z	
Cerebellum Posterior Lobe		L	94	−15	−78	−51	5.75
Cerebellum Posterior Lobe		R	40	6	−63	−54	5.30
Cerebellum Posterior Lobe		R	30	42	−66	− 54	5.09
Cerebellum Posterior Lobe		R	49	6	−54	−39	5.25
Brainstem		R	117	18	−18	− 39	6.46
Brainstem		L	30	−6	− 15	−18	5.55
Inferior Temporal Gyrus	20	R	33	60	−24	−21	5.22
Middle Temporal Gyrus		L	40	−66	−45	−18	7.72
Superior Temporal Gyrus	39	L	47	−57	−57	21	6.02
Parahippocampa Gyrus	30	R	42	15	−33	−6	5.52
Precuneus	31	L	42	− 3	−51	30	4.56
Superior Frontal Gyrus	6	L	38	−12	9	72	6.59
Superior Frontal Gyrus	6	R	30	9	0	72	6.00

The anatomical locations, approximate Brodmann areas (BA) and MNI coordinates that corresponded to the t-values of the representative peaks within each cluster were reported. A positive t-value means that fALFF was increased in this brain region after acupuncture treatment. All regions reached a voxel-level significance threshold of *P* < 0.001, FWE correct *P* = 0.05, cluster size> 30

**Table 8:** The altered fALFF in brain regions of M-Acu group after 5-weeks acupuncture

Brain regions	BA	Side	Cluster size	MNI			t-value
				X	Y	Z	
Cerebellum Posterior Lobe		R	50	15	−33	−60	5.03
Parahippocampa Gyrus	36	L	38	−27	−36	−15	5.70
Middle Temporal Gyrus	21/22	L	261	−54	−57	0	6.82
Superior Temporal Gyrus							
Superior Temporal Gyrus		R	33	45	−18	−9	4.96
Middle Temporal Gyrus	37	R	54	48	−39	−9	6.12
Superior Temporal Gyrus		R	73	45	−39	9	6.67
Middle Temporal Gyrus		L	42	−45	−78	9	5.09
Middle Frontal Gyrus		L	119	−39	30	24	5.69
Inferior Frontal Gyrus	6/9	L	525	−45	3	30	7.60
Precentral Gyrus							
Postcentral Gyrus							
Precentral Gyrus		R	43	42	−3	30	4.93
Postcentral Gyrus	3/4	R	66	60	−21	39	5.64
Precentral Gyrus							
Inferior Parietal Lobule	40	L	371	−48	−57	33	6.00
Angular Gyrus							
Supramarginal Gyrus							
Postcentral Gyrus							
Cuneus	18/19	L	370	−21	−51	39	5.65
Precuneus							
Superior Parietal Lobule							
Precuneus	39	R	67	33	−72	42	5.02

The anatomical locations, approximate Brodmann areas (BA) and MNI coordinates that corresponded to the t-values of the representative peaks within each cluster were reported. A positive t-value means that fALFF was increased in this brain region after acupuncture treatment. All regions reached a voxel-level significance threshold of *P* < 0.001, FWE correct *P* = 0.05, cluster size> 30

**Table 9:** The altered fALFF in brain regions of N-Acu group after 5-weeks acupuncture

Brain regions	BA	Side	Cluster size	MNI			t-value
				X	Y	Z	
Superior Frontal Gyrus		L	34	−21	63	3	4.63
Superior Frontal Gyrus	10	R	364	21	60	6	5.38
Medial Frontal Gyrus							
Anterior Cingulate							
Insula	13	L	30	−33	21	6	4.16
Middle Frontal Gyrus	46	L	33	−45	36	21	4.49
Middle Frontal Gyrus	46	R	83	42	24	24	4.44
Inferior Frontal Gyrus	9	R	51	57	6	36	5.09

The anatomical locations, approximate Brodmann areas (BA) and MNI coordinates that corresponded to the t-values of the representative peaks within each cluster were reported. A positive t-value means that fALFF was increased in this brain region after acupuncture treatment. All regions reached a voxel-level significance threshold of *P* < 0.001, FWE correct *P* = 0.05, cluster size> 30

#### The different altered fALFF regions of PI patients in different groups

Compared the increased fALFF in brain regions between the S-Acu group and N-Acu group, we found the increased fALFF in right anterior cingulate and left insula of N-Acu group were higher than that of S-Acu group. The increased fALFF in left cerebellum posterior lobe and right brainstem of S-Acu group were higher than that of N-Acu group (*P* < 0.005, FWE correct *P* = 0.05, cluster size> 30) (Table 10).

**Table 10:** The different altered fALFF regions of different groups after acupuncture treatment

Group	Brain regions	BA	Side	Cluster size	MNI			t-value
					X	Y	Z	
S vs N	Anterior Cingulate	25	R	51	3	18	−9	−5.12
	Insula		L	168	−15	30	15	−6.93
	Cerebellum Posterior Lobe		L	84	−15	−57	−57	4.51
	Brainstem		R	38	6	−24	−39	4.56
S vs M	Parahippocampa Gyrus		L	35	−27	− 36	−15	−5.18
	Superior Temporal Gyrus Middle Temporal Gyrus	22	L	348	−57	−18	−3	− 5.83
	Superior Temporal Gyrus		R	31	45	−39	9	−5.02
	Lentiform Nucleus		R	91	18	9	0	−4.92
	Middle Frontal Gyrus		L	37	−30	42	18	−5.09
	Middle Frontal Gyrus		L	33	−36	15	42	−4.69
	Middle Frontal Gyrus	6	L	50	−27	0	66	−4.66
	Precentral Gyrus		L	126	−51	0	12	−5.40
	Inferior Frontal Gyrus							
	Inferior Frontal Gyrus	9	L	46	−48	3	27	−5.58
	Postcentral Gyrus	40	L	152	−48	−27	39	−4.61
	Inferior Parietal Lobule							
	Superior Parietal Lobule	7	L	55	−33	−69	48	−5.09
	Anterior Cingulate		L	62	−24	27	24	−5.32
	Supramarginal Gyrus	40	L	110	−54	−48	36	−5.73
	Angular Gyrus							
	Precuneus	39	R	33	36	−69	42	−4.59

The anatomical locations, approximate Brodmann areas (BA) and MNI coordinates that corresponded to the t-values of the representative peaks within each cluster were reported. *S vs N* S-Acu group vs N-Acu group. A positive t-value means that the increased fALFF in this brain region of S-Acu group was higher than that of N-Acu group. *S vs M* S-Acu group vs M-Acu group. A negative t-value means that the increased fALFF in this brain region of S-Acu group was lower than that of M-Acu group. All regions reached a voxel-level significance threshold of *P* < 0.005, FWE correct *P* = 0.05, cluster size> 30

Compared the increased fALFF in brain regions between the S-Acu group and M-Acu group, we found the increased fALFF in left parahippocampa gyrus, bilateral superior temporal gyrus, left middle temporal gyrus, right lentiform nucleus, left middle frontal gyrus, left inferior frontal gyrus, left precentral gyrus, left postcentral gyrus, left inferior parietal lobule, left superior parietal lobule, left anterior cingulate, left supramarginal gyrus, left angular gyrus and right precuneus of M-Acu group were higher than those of S-Acu group (*P* < 0.005, FWE correct *P* = 0.05, cluster size> 30) (Table 10).

## Discussion

According to the Traditional Chinese Medicine (TCM) theory, acupuncture play a therapeutic role by stimulating at the acupoints, and producing special needling/tactile somatosensory which is called De-Qi. Patients experienced De-Qi as multiple unique sensations at the needle site and surrounding regions. MASS scale was used as an appropriate method to measure De-Qi sensation [26]. In our study, there was no significant difference of the MASS scale among three groups.

However, not limited to this, the placebo effect also plays a role in treatment process. Research suggested that the sham acupuncture without somatosensory tactile stimulation could still improve the clinical symptoms [27]. The primary outcomes of our study suggested that clinical symptoms were improved in all groups after 5 weeks acupuncture treatment, including the N-Acu group (sham point). However, the decreased PSQI score of S-Acu was higher than that of N-Acu group. In addition, the PSG results suggested that the TST (*P* = 0.020) and R (*P* = 0.001) of S-Acu group were significant increased after 5-weeks acupuncture. Although the TST and R were also increased in N-Acu group, no significant difference was found after 5-weeks acupuncture. Therefore, we suggested that acupuncture could improve the symptoms and patient’s subjective experience, and the real acupoints were more effective than sham points.

Then the efficacy of the two acupuncture regimens, single acupoint (HT-7) and combination of multi-acupoints (HT-7, SP-6 and GV-20) were compared, the primary outcomes suggested that the decreased AIS score and PSQI score of M-Acu group were slightly higher than those of S-Acu group, although there was no significant difference between the two groups. In addition, the PSG results revealed that the TST and REM in S-Acu group were significantly increased. And in M-Acu group, the TST, SE, N3 and REM were significantly increased, while the AI and WASO were significantly decreased. REM sleep engage brain development functions to facilitate the formation and consolidation of certain types of memory and learning processes [28]. The prolongation of the REM stage is evidence that support the acupuncture at a single acupoint or a combination of multi-acupoints both have a positive effect on PI patients. Furthermore, the N3 time was prolonged in M-Acu group, which was not observed in S-Acu group. And the increased TST of M-Acu group was significantly longer than that of S-Acu group. Above all, these findings of primary and secondary clinical outcomes suggested that the combination of multi-acupoints might enhance clinical efficacy.

In order to further explore the mechanism of acupuncture therapy, we designed the resting state fMRI study to observe the brain activity of PI patients before and after 5-weeks acupuncture. First, we studied the relationship between the brain activity (fALFF value) and sleep quality (AIS score), we found that some brain regions had higher fALFF levels when AIS scores were lower. These regions included the right cerebellum posterior lobe [29, 30], temporal gyrus (left middle temporal gyrus [30], left superior temporal gyrus [15]), left extra-nuclear, right anterior cingulate [31], frontal gyrus (bilateral inferior frontal gyrus [30], left middle frontal gyrus [29, 30], bilateral superior frontal gyrus [29]), parietal lobule (right precentral gyrus, left supramarginal gyrus, left inferior parietal lobule [29, 30], left superior parietal lobule, left precuneus [29, 31]). Researches suggested that differences in glucose metabolism rates or ALFF between insomnia patients and healthy controls in these brain regions [29–31]. Furthermore, our results suggested that acupuncture could modulate the ALFF value of these regions. And the number of regulated brain areas in M-Acu group is much higher than that in S-Acu group or N-Acu group.

Compared with the S-Acu group, the increased fALFF of the N-Acu group in anterior cingulate and insula were more obvious. Studies have shown that real acupuncture could exhibit greater activation in the anterior cingulate gyrus and insula regions than phantom acupuncture without somatosensory tactile stimulation [27]. However, sham acupuncture in our trial would produce pain stimulation, and MASS results showed that sharp pain scores were slightly higher than that in the S-Acu group, although there was no statistical difference between the two groups. Therefore, we speculated that the increased fALFF in the anterior cingulate gyrus and insula may be caused by pain stimulation. And the increased fALFF in the cerebellum posterior lobe and brainstem may be caused by the stimulating on specific acupoint of S-Acu group.

Compared with the S-Acu group, we found the increased fALFF in parahippocampa gyrus, superior temporal gyrus, middle temporal gyrus, lentiform nucleus, middle frontal gyrus, inferior frontal gyrus, precentral gyrus, postcentral gyrus, inferior parietal lobule, superior parietal lobule, anterior cingulate, supramarginal gyrus, angular gyrus and precuneus of M-Acu group were more obvious.

The brain activity of some brain regions which were regulated in M-Acu group, including superior temporal gyrus [15], middle temporal gyrus [30], middle frontal gyrus [29, 30], inferior frontal gyrus [30], superior parietal lobule, inferior parietal lobule [29, 30], anterior cingulate [31], supramarginal gyrus, precuneus [29, 31], were varied between insomnia patients and healthy controls. And our study also suggested that brain activity in these brain regions were negatively correlated with AIS scores. Therefore, we considered that for primary insomnia, multi-acpoints stimulation might produce a greater therapeutic effect.

## Conclusion

In our clinical trials, acupuncture has been proven to be beneficial for PI patients, and the combination of multi-acupoints could improve its efficacy. The relationship between the brain activity (fALFF value) and sleep quality (AIS score) suggested that the brain activity of certain regions was higher when sleep quality was better, while part of these regions could be regulated by acupuncture on single acupoints/sham point/combination of multi-acupoints. The effect of multi-acupoints group on regulating the brain activity was most obvious, which may be the possible mechanism of that multi-acupoints stimulating could improve the acupuncture efficacy.

## Limitations

The present trial aims to select a more effective acupoint scheme for insomnia in clinical acupuncture treatment. The different efficacy between a single acupoint and a combination of acupoints were mainly compared in the present trial. However, the present data remains preliminary, and the comparison of efficacy produced by these different single acupoints were based on the previous studies, which have not been investigated in the present trial. More studies are needed to compare the efficacy difference among different single acupoints.

## Data Availability

The datasets generated and/or analyzed in the present study can be available from the corresponding author upon reasonable request.

## Abbreviations

ACC: Anterior cingulate cortex
AI: Arousal index
AIS: Athens insomnia scale
ALFF: Amplitude of low-frequency fluctuation
BA: Brodmann area
BOLD: Blood oxygenation level dependent
DPARSF: Data processing assistant for resting-state fMRI
FA: Flip angle
FOV: Field of view
FWE: Family wise error rate
MASS: Massachusetts General Hospital acupuncture sensation scales
MNI: Montreal neurological institute
NREM: Non-rapid eye movement
PI: Primary insomnia
PSG: Polysomnography
PSQI: Pittsburgh sleep quality index
REM: Rapid eye movement
Rs-fMRI: Resting state functional magnetic resonance imaging
SAS: Self-rating anxiety scale
SDS: Self-rating depression scale
SE: Sleep efficient
SOL: Sleep onset latency
SPM: Statistical parametric mapping toolbox
TR: Repetition time
TE: Echo time
TST: Total sleep time
WASO: Wake after sleep onset
